# High-Resolution Phase-Based Ranging Using Inverse Fourier Transform in an Iterative Bayesian Approach

**DOI:** 10.3390/s24206758

**Published:** 2024-10-21

**Authors:** Jan Mazur

**Affiliations:** Faculty of Electronics Photonics and Microsystems, Wroclaw University of Science and Technology, Wybrzeze Wyspianskiego 27, 50-370 Wroclaw, Poland; jan.mazur@pwr.edu.pl

**Keywords:** phase-based ranging, distance estimation, localization, high-resolution Fourier transform, Bayesian approach, Bluetooth Low Energy, channel sounding

## Abstract

This article proposes an algorithm that determines the distance between two transceivers based on phase information collected in a specific frequency range. Even though we have focused on BLE technology, we do not necessarily adhere strictly to this standard regarding the procedures used to obtain phased samples. We assume that phase samples are given and propose an algorithm using a Bayesian approach to find delays in a multi-path environment. Analyzing these delays allows for determining the distance between both transceivers. We show several examples confirming the high accuracy and resolution of the proposed algorithm. Finally, we conclude with some pros and cons of the proposed solution, suggesting its use in such applications as, for example, virtual acoustics.

## 1. Introduction

Localization is one of the biggest challenges in many of today’s applications, from military object positioning through global and local asset tracking and vehicle navigation, to localizing people and assets indoors and outdoors [1,2,3,4,5]. Because of the broad range of these applications, many dedicated technologies arose to fulfill relatively different requirements concerning localization in those applications [1,2]. These technologies range from radar or radar-like systems through GPS and other global navigation systems, to local cellular systems and small beacon-based or similar systems [1], with Bluetooth Low Energy (BLE) [6,7] and ultra-wide-band (UWB) [8] technologies being the most popular. Even if the latter seems to be the most accurate technology of localization at present, we will focus on BLE and its potential in the area of localization or, more precisely, distance estimation, which is a core part of multilateration-based localization techniques. A typical setup of such systems is a set of so-called beacons or anchors of known positions that transmit a constant tone in a given range of frequencies to the transceiver attached to the object that is to be localized. This transceiver can retransmit received signals in a given way back to the anchors or it can transmit its signals to them. There are several techniques in this setup for processing these signals to find an object’s position.

It seems that three techniques cover the majority of all applications being used. The first and the simplest is the RSSI (Received Signal Strength Index), in which the anchors measure the strength of the signal received from the transmitter attached to the object, and using a multilateration procedure, they estimate the position of the object in 2D or 3D [5,9]. Due to the non-uniformity of the field (fading channel), this technique is not very accurate (5–10 m).

The second technique that can be used is called ToF (time of flight) with some variations, in which the time of flight between the transmitter (anchor) and the receiver (object) is measured [10]. Using this time, the distance between them can be measured. Having at least three distances from the object to the anchors, it is possible to find the object’s position. This technique is usually more accurate than the RSSI (3–5 m). The third technique, which seems to be the most accurate (0.5–4.0 m), is based on phase measurements and is commonly known as PBR (phase-based ranging) [11,12,13,14,15,16]. There are, ultimately, two variants of phase-based ranging. One of them, simpler but less accurate, is based on analyzing the phase slope as a function of (angular) frequency. The second is based on the Fourier inversion of complex exponentials (in a general case) whose arguments are phase data [17].

The idea of using phase samples comes from the fact that a single tone (carrier) transmitted from the transmitter arrives at the receiver with a phase that depends on the distance between them. Assuming that the local oscillators of both the transmitter and receiver are synchronized, the only phase difference comes from the distance and can be relatively easily measured. Knowing the phase samples for at least two frequencies is enough to find the slope dϕ/dω, which is equal to the time the wave travels from the transmitter to the receiver. Unfortunately, this method—even if we can gather phase samples for more than two frequencies—is prone to errors due to non-linearities of the phase function in case of the fading channel (reflections) and a relatively narrow bandwidth. Additionally, it is ill-conditioned, in that, small errors in phase or frequency measurement can generate large errors in the result. Much better results can be obtained using inverse Fourier transform. In this technique, the phase function does not have to be unwrapped directly. Instead, the inverse Fourier transform is performed, which results in “time-lines” (similarly, when we find spectral lines, we perform the Fourier transform of complex exponentials in the time domain).

The main problem of this method is usually a small number (about a dozen or several dozen) of phase samples, which is the reason for the necessary choice of either the long range or high resolution (accuracy of distance/time measurement), whereby the former depends on the frequency resolution and the latter on the analysis bandwidth. To increase the resolution of the inverse Fourier transform, the phase series can be zero-padded. Still, this solution does not generate any new information on the phase function, and the resolution of the resulting series is proportional to the number of non-zero samples.

To overcome this problem, we propose a method of finding high-resolution inverse Fourier transform which—under certain circumstances—can give very accurate and stable results. By the term “high resolution”, we mean that the length of the output series of the algorithm is longer than the length of the input series while covering the same time range, and we can still distinguish between two consecutive bins.

## 2. Distance Estimation Between Two Transceivers

### 2.1. Problem Formulation

Here, we want to describe the problem of the phase-based distance estimation between two (or more) transceivers in the presence of a noisy fading channel, which seems to be the most common case in indoor and outdoor environments. For those readers who are not very familiar with phase-based ranging, a short introduction is given in Appendix A.

We will show what this “phase” is and how it can be used for distance estimation in the case of synchronized and unsynchronized transceivers, as it is in Bluetooth Low Energy (BLE) [6].

**Figure 1:**
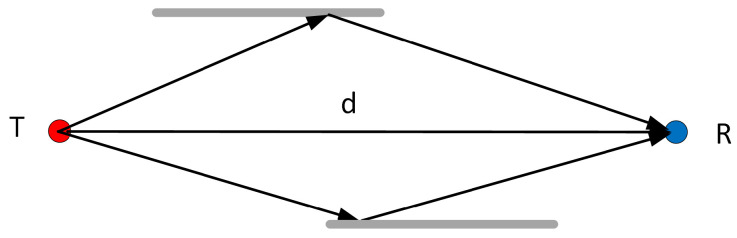
Transmitter (T) and receiver (R) setup. LOS and NLOS (bouncing against obstacles) signals.

Let us assume that we have a transmitter (T) and a receiver (R) with a distance of *d* between them (see Figure 1), which are capable of transmitting and receiving pure tones (carriers) in a certain number of channels.

Let x(t) be the signal transmitted from the transmitter (T) to the receiver (R). The signal may travel directly from T to R (LOS signal) and/or may bounce against obstacles such as walls or furniture (NLOS signal), creating attenuated and delayed copies of itself at R; so, the signal can be described at the receiver as

(1) $$y\left(t\right)=\sum_{m=1}^{M}a_{m}x\left(t-t_{m}\right)=x\left(t\right)*\sum_{m=1}^{M}a_{m}\delta \left(t-t_{m}\right),$$

where ∗ denotes the convolution operator and δ(t) is the Dirac delta distribution. Note that the time *t* for *x* and *y* is the same time, which means that both the transmitter and the receiver use the same time. In other words, they have to be synchronized for the above to hold.

Now, let X(ω)=F{x(t)} and Y(ω)=F{y(t)} be the Fourier transforms of their respective signals. From the shifting property of a Fourier transform, we know that

(2) $$F\left\{x\left(t-\tau \right)\right\}=e^{-j\tau \omega }X\left(\omega \right).$$

Applying this property to y(t), we obtain

(3) $$Y\left(\omega \right)=F\left\{y\left(t\right)\right\}=F\left\{\sum_{m=1}^{M}a_{m}x\left(t-t_{m}\right)\right\}=X\left(\omega \right)\sum_{m=1}^{M}a_{m}e^{-jt_{m}\omega }.$$

Dividing both sides of the above equation by X(ω), we obtain the Fourier transform of the channel from T to R:

(4) $$H_{TR}\left(\omega \right)=Y\left(\omega \right)/X\left(\omega \right)=\sum_{m=1}^{M}a_{m}e^{-jt_{m}\omega },\;\;\;\;\omega :\;X\left(\omega \right)\neq 0,$$

where $H_{TR}$ is the channel transmittance from the transmitter to the receiver.

We can see that the RHS of the above equation is the sum of complex exponentials, each of which is characterized (parameterized) by one delay parameter $t_{m}$. Taking the inverse Fourier transform of this sum, i.e.,

(5) $$F^{-1}\left\{\sum_{m=1}^{M}a_{m}e^{-jt_{m}\omega }\right\}=\sum_{m=1}^{M}a_{m}\delta \left(t-t_{m}\right),$$

we obtain the series of spikes or “time-lines” at the points of each delay, which is exactly the RHS of (1). We call them “time-lines” to differentiate them from “spectral lines” even if, more formally, they should be called “spectral lines in the time domain”. The position of the first spike designated as $t_{1}$ can be interpreted as the delay of the signal x(t) transmitted by a transmitter when received by the receiver in the LOS case. The distance between them can be estimated as $d=ct_{1}$. Notice that there is no guarantee that the first delay corresponds to the LOS case. It is only the shortest flight time of the wave between T and R.

However, the method described above has two significant limitations. First, as mentioned above, the transmitter and receiver must be synchronized to correctly interpret the set of $t_{m}$ values for all m=1..M. Second, the receiver must know the signal x(t) or its Fourier transform. To overcome these limitations, y(t) is sent back to the transmitter. Let

(6) $$v\left(t\right)=\sum_{n=1}^{N}b_{n}y\left(t-\tau_{n}\right)=y\left(t\right)*\sum_{n=1}^{N}b_{n}\delta \left(t-\tau_{n}\right)$$

be the signal received by the transmitter, where $b_{n}$ are attenuations of the delayed signals and $\tau_{n}$ are values of those delays for all n=1..N. By performing the Fourier transform of both sides, we obtain

(7) $$H_{RT}\left(\omega \right)=V\left(\omega \right)/Y\left(\omega \right)=\sum_{n=1}^{N}b_{n}e^{-j\tau_{n}\omega },\;\;\;\;\omega :\;Y\left(\omega \right)\neq 0,$$

where $H_{RT}$ is the channel transmittance from the receiver to the transmitter. By combining (4) and (7), we obtain the transmittance of the channel in both directions:

(8) $$H_{TRT}\left(\omega \right)=\frac{V(\omega )Y(\omega )}{Y(\omega )X(\omega )}=\sum_{n=1}^{N}b_{n}e^{-j\tau_{n}\omega }\sum_{m=1}^{M}a_{m}e^{-jt_{m}\omega },\;\;\;\;\omega :\;X\left(\omega \right)\neq 0.$$

This can be rewritten in a reordered form as

(9) $$H_{TRT}\left(\omega \right)=\sum_{m=1}^{M}\sum_{n=1}^{N}a_{m}b_{n}e^{-j(\tau_{n}+t_{m})\omega }=\sum_{p=1}^{P}c_{p}e^{-jt_{p}\omega },\;\;\;\;\;m=1..M,n=1..N,$$

where $p=1..P=arg(sort\left({\left\{t_{m}+\tau_{n}\right\}}_{m=1,n=1}^{M,N}\right))$ and $c_{p}=a_{m}b_{n}$.

Since the transmitter knows x(t), it can correctly determine the values of $exp(-jt_{p}\omega )$. However, note that we assumed the immediate sending back of y(t) from the receiver to the transmitter, which is not true. Nevertheless, this idea stands behind the development of the procedure for determining phase samples in the BLE standard.

We will not go through the details of the abovementioned procedure because it is thoroughly described in the BLE standard documentation [6,18]. Instead, we will briefly describe the main steps leading to the result, i.e., the series of phase samples. Solutions more embedded in the BLE standard can be found, for instance, in [11,14]. The Bluetooth Low Energy (BLE) standard V 5.0 [6] defines 40 2 MHz-wide channels with center frequencies (carriers) $f_{n}^{\left(c\right)}=2402+2n\;\mathrm{MHz},\;\;\;n=1..N=40$. It is worth noticing that in the yet-to-come version 5.4 [18], it defines 79 1 MHz-wide channels with center frequencies $f_{n}^{\left(c\right)}=2402+1n\;\mathrm{MHz},\;\;\;n=1..N=79$. In this article, we will roughly assume what version 5.0 defines.

The first step is to generate a series of IQ samples on the Initiator side:

(10) $$x_{IQ}\left(t\right)=e^{j\omega_{0}t},$$

where $\omega_{0}$ is the angular frequency of the modulating series. In the next step, this signal modulates the carrier of angular frequency: $\omega_{k}^{\left(c\right)}$, giving

(11) $$x\left(t\right)=x_{IQ}\left(t\right)e^{j\omega_{k}t}=e^{j(\omega_{0}+\omega_{k}^{\left(c\right)})t},$$

which, when propagating through space, is attenuated and delayed, creating

(12) $$y\left(t\right)=ax\left(t\right)*\delta \left(t-\tau_{IR}\right)=ax\left(t-\tau_{IR}\right)=ae^{j\left(\omega_{0}+\omega_{k}^{\left(c\right)}\right)\left(t-\tau_{IR}\right)}$$

on the Reflector side. This signal is then demodulated to the IQ signal:

(13) $$y_{IQ}\left(t\right)=y\left(t\right)e^{-j(\omega_{k}^{\left(c\right)}t+\phi_{IR})}=ae^{j\left(\omega_{0}+\omega_{k}^{\left(c\right)}\right)\left(t-\tau_{IR}\right)}e^{-j(\omega_{k}^{\left(c\right)}t+\phi_{IR})}.$$

Putting $\theta_{IR}=-\left(\omega_{0}+\omega_{k}^{\left(c\right)}\right)\tau_{IR}$ gives

(14) $$y_{IQ}\left(t\right)=ae^{j(\omega_{0}t+\theta_{IR}-\phi_{IR})}.$$

Comparing $y_{IQ}\left(t\right)$ with $x_{IQ}\left(t\right)$, we notice the additional phase terms $\theta_{IR}$ and $\phi_{IR}$. The former is related to the distance between the Initiator and the Reflector and the latter comes from the phase shift between their local oscillators. At this point, the Reflector sends the received signal back to the Initiator. To distinguish the direction the signal travels, we changed incoming $y_{IQ}\left(t\right)$ into outgoing $v_{IQ}\left(t\right)$:

(15) $$v_{IQ}\left(t\right)=y_{IQ}\left(t\right),$$

which modulates the carrier of the same frequency as the Initiator’s carrier, giving

(16) $$v\left(t\right)=ae^{j(\omega_{0}t+\theta_{IR}-\phi_{IR})}e^{j\omega_{k}^{\left(c\right)}t}.$$

Similarly to x(t), the above is attenuated and delayed in the air, creating

(17) $$u\left(t\right)=bv\left(t\right)*\delta \left(t-\tau_{RI}\right)=bv\left(t-\tau_{RI}\right)=bae^{j(\omega_{0}\left(t-\tau_{RI}\right)+\theta_{IR}-\phi_{IR})}e^{j\omega_{k}^{\left(c\right)}\left(t-\tau_{RI}\right)}$$

on the Initiator side. At the end, u(t), when demodulated, gives IQ samples on the Initiator’s side

(18) $$u_{IQ}\left(t\right)=u\left(t\right)e^{-j(\omega_{k}^{\left(c\right)}t+\phi_{RI})}=bae^{j(\omega_{0}\left(t-\tau_{RI}\right)+\theta_{IR}-\phi_{IR})}e^{j\omega_{k}^{\left(c\right)}\left(t-\tau_{RI}\right)}e^{-j(\omega_{k}^{\left(c\right)}t+\phi_{RI})}.$$

Now, putting $\theta_{RI}=-\left(\omega_{0}+\omega_{k}^{\left(c\right)}\right)\tau_{RI}$, and simplifying the above expression, we obtain

(19) $$u_{IQ}\left(t\right)=bae^{j(\omega_{0}t+\theta_{IR}+\theta_{RI}-\phi_{IR}-\phi_{RI})}.$$

Notice that $\phi_{IR}+\phi_{RI}=0$ by their definitions, so that

(20) $$u_{IQ}\left(t\right)=bae^{j(\omega_{0}t+\theta_{IR}+\theta_{RI})}=bae^{j(\omega_{0}t+\theta )},$$

where $\theta =\theta_{IR}+\theta_{RI}$ is the phase shift related to the distance along which the wave travels from the Initiator to the Reflector and back. This value is estimated in each of *N* channels Δ*F*MHz-wide, resulting in a series of phase data

(21) $$\theta =[\theta_{1},\theta_{2},\ldots \theta_{n}\ldots \theta_{N}],$$

where N=40 for ΔF=2 MHz or N=80 for ΔF=1 MHz, depending on the BLE version chosen. Now, let H(n) be a complex exponential of the form:

(22) $$H\left(n\right)=\left[H_{1}e^{j\theta_{1}},H_{2}e^{j\theta_{2}},\ldots H_{n}e^{j\theta_{n}}\ldots H_{N}e^{j\theta_{N}}\right],$$

where $e^{j\theta_{n}}=e^{-j\left(\tau_{IR}+\tau_{RI}\right)\omega_{n}}$, or, in a more general case of many delays—if we recall (9)—we obtain

(23) $$e^{j\theta_{n}}=\sum_{p=1}^{P}c_{p}e^{-jt_{p}\omega_{n}}$$

so that the IDFT of (22) can be expressed as

(24) $$h\left(m\right)=\sum_{n=0}^{N-1}\sum_{p=1}^{P}c_{p}e^{-jt_{p}\omega_{n}}e^{j\frac{2\pi }{N}mn}.$$

Moreover, by putting $\omega_{n}=2\pi n\Delta F$ and $t_{p}=m_{p}\Delta T$, we obtain

(25) $$h\left(m\right)=\sum_{n=0}^{N-1}\sum_{p=1}^{P}c_{p}e^{-jm_{p}\Delta T2\pi n\Delta F}e^{j\frac{2\pi }{N}mn}.$$

After reordering sums, we eventually obtain

(26) $$h\left(m\right)=\sum_{p=1}^{P}c_{p}\sum_{n=0}^{N-1}e^{j2\pi n(m-m_{p})/N}=\sum_{p=1}^{P}c_{p}\delta \left(m-m_{p}\right),\;\;\;m=1..N.$$

With the frequency domain defined as above, the period of the first basis function of the Fourier transform is $T_{max}=1/\Delta F$ long or, in terms of distance in space, it is $D_{max}=cT_{max}=300$ m for 1MHz-wide channels and $D_{max}=150$ m for 2MHz-wide channels, where $c=3\times 10^{8}$ m/s is the speed of light.

The time domain resolution is then simply $\Delta T=T_{max}/N=12.5$ ns, or it can be any other value derived from it, and the spatial resolution is ΔD=cΔT=3.75 m. In many applications, a resolution of about 4 m is not enough. To improve the spatial resolution, we need to (1) decrease $T_{max}$, as $\Delta T=T_{max}/N$, keeping *N* constant; (2) increase $F_{max}=1/\Delta T$; or (3) increase *N*, keeping $T_{max}$ constant. We have chosen the last option for further consideration and we will briefly discuss this choice in the following section.

Because of a relatively limited number of available phase samples, the problem can be formulated as follows: *given the low-resolution data in the frequency domain, find a high-resolution result in the time domain.* This result will be a series of delta functions at positions determined by the delays of all (or only significant) paths of the signal transmitted from the Initiator to the Reflector and back. We assume that the shortest delay corresponds to the LOS path delay, which directly determines the distance we are looking for, unless there is no LOS path.

### 2.2. Solution of the Problem

Here, we present a solution to the problem we formulated at the end of the previous section. Phase samples might be gathered, for example, in a procedure of channel sounding (or similar) when a continuous tone is transmitted from the Initiator to the Reflector and back in a certain number of channels—one channel at a time; but, as a matter of fact, the way the phase samples were obtained is not important. We will assume they are given and that they were obtained using a model of the fading channel described by (9) so that the expected result can be presented in the form of (26). To solve the problem we formulated in the previous section, we should extend the bandwidth as its reciprocal is directly related to resolution in the time domain, which in turn (knowing the speed of the wave), directly translates to the resolution in space. However, bandwidth extension is often impossible because the transmission usually takes place in a specific standard (like BLE) that strictly defines the bandwidth. The fixed bandwidth with a fixed number of channels implies the fixed range, which is a reciprocal of the distance between the channels. Increasing the number of channels would then increase the range, keeping the resolution in time/space constant. So, increasing the resolution in time is only possible by applying a kind of interpolation in the time domain.

One obvious way to perform this is to create a zero-padded series including the series of phase data. However, it does not allow for the incorporation of any additional knowledge into the result. Therefore, we propose an iterative algorithm of distance (time delay) estimation using the Bayesian approach that uses the available phase data more efficiently, assuming the (inverse) transform is sparse in the time domain (i.e., there are just a few dominant reflections). This “sparseness” seems to be quite a reasonable requirement as we are looking for “spikes” (1) and (5), which are transforms of the periodic complex exponentials in the Fourier domain that naturally arise as functions of the linear phase argument.

The proposed algorithm was originally developed to solve the spectrum estimation problem in which complex time domain exponentials are transformed into Fourier domain delta functions. However, the phase-based distance estimation problem is exactly the opposite. Thus, we have two approaches to choose from: modify this algorithm to implement IDFT or (much simpler) use this algorithm directly, which means that we perform the DFT and then use the following simple Fourier transform property:

(27) $$\int X\left(\omega \right)e^{j\omega t}d\omega =\int X\left(\omega \right)e^{-j\omega (-t)}d\omega =x\left(-t\right)$$

to find the proper result. In other words, instead of performing IDFT, we will perform DFT and invert the (time) domain of the resulting transform.

The former approach is more formal and seems to be more elegant. However, the latter is simpler and it provides the reader with the possibility to look into the original paper [19] where this algorithm is thoroughly explained and discussed and find more details on it without the necessity of rewriting the proposed algorithm back to its original version. More on the Bayesian approach in signal and image processing can be found in [20]. Below, we will briefly present the idea and the most important steps of the proposed algorithm, and then show how to use its implementation in the distance estimation task.

Let us assume that x(n) of length *N* is a signal composed of a certain number of complex exponentials in the time domain; we want to find its high-resolution Fourier transform X(m) of length M>N, so that

(28) $$x=FX,\;\;\;\;\;\;\mathrm{with}\;\;\;F_{nm}=\left(1/N\right)exp\left(j2\pi nm/M\right)),$$

where *X* is a column vector of size *M* containing Fourier transform coefficients of signal *x* of size *N*, and matrix $F_{N\times M}$ contains the exponential terms defining Fourier transformation.

For the algorithm to work, we need to define a data model; so, we assume that the data are contaminated with normally distributed noise $N(0,\sigma_{n}^{2})$. In addition, we need to define the prior distribution of *X* conditional on a parameter $\sigma_{X}$ as well as the distribution of *x* conditional on *X* and both $\sigma_{X}$ and $\sigma_{n}$ parameters. Then, we need to use the known Bayes rule to find the posterior probability of *x* conditional on $X,\sigma_{X}$ and $\sigma_{n}$, i.e.,

(29) $$p\left(X|x,\sigma_{X},\sigma_{n}\right)=\frac{p\left(x|X,\sigma_{n}\right)p\left(X|\sigma_{X}\right)}{p(x|X,\sigma_{X},\sigma_{n})},$$

where

(30) $$p\left(x|X,\sigma_{n}\right)={\left(2\pi \sigma_{n}^{2}\right)}^{-(N-1)/2}exp\left(-||x-{FX||}^{2}/\left(2\sigma_{n}^{2}\right)\right)$$

is the conditional distribution of *x* (data) and

(31) $$p\left(X|\sigma_{X}\right)=\prod_{m=1}^{M}p\left(X_{m}|\sigma_{X}\right)$$

is the prior distribution of *X* conditional on $\sigma_{X}$. This prior is assumed to be the Cauchy distribution:

(32) $$p\left(X_{m}|\sigma_{X}\right)\propto {\left(1+\frac{X_{m}X_{m}^{*}}{2\sigma_{X}^{2}}\right)}^{-1},$$

where $X_{m}$ is the *m*-th Fourier coefficient. Combining this prior with the data likelihood given by (30), the cost function (given in [19]) can be found, which is then used to derive equations that allow for finding the best X in the following three-step iterative procedure:

(33) $$\begin{array}{ccc} b_{\left(k-1\right)} & = & {\left[\lambda I_{n}+FQ_{\left(k-1\right)}F^{H}\right]}^{-1}x\\ X_{\left(k\right)} & = & Q_{\left(k-1\right)}F^{H}b_{\left(k-1\right)}\\ Q_{(k)mm} & = & 1+X_{(k)m}X_{(k)m}^{*}/\left(2\sigma_{X}^{2}\right),\;\;\;\;m=0..M-1, \end{array}$$

where *b* is an auxiliary vector, $I_{n}$ is the identity matrix of size n×n, and $\lambda =\sigma_{n}^{2}/\sigma_{X}^{2}$ is a trade-off. For λ>0, parameter $\sigma_{X}$ controls the ‘amount of sparseness’, but this can be affected by $\sigma_{n}$ if the latter is large enough. The values of $Q_{\left(0\right)}$ are computed using $X_{\left(0\right)}$ of size *M*, which is a zero-padded DFT of *x*. Notice that in the third step of the above procedure, the diagonal elements of the diagonal matrix Q of size M×M implement the prior defined in (32), while in the second step, *Q* is used to modify *X*. This way, the values of *X* are iteratively modified to promote the bins that match *x* best.

**Figure 2:**
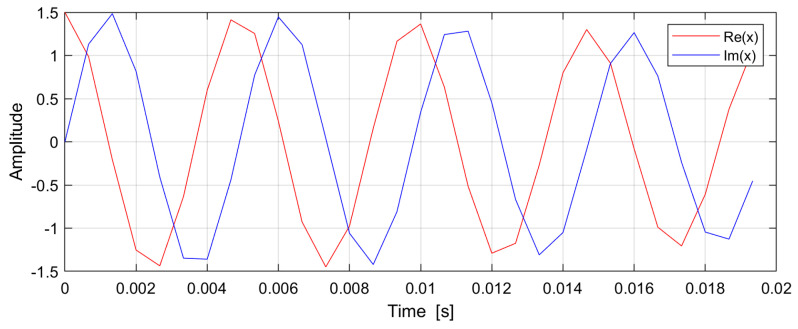
Example signal (30 samples, SNR = 80 dB): real (red) and imaginary (blue) part of a signal consisting of two frequencies 200 Hz and 210 Hz.

**Figure 3:**
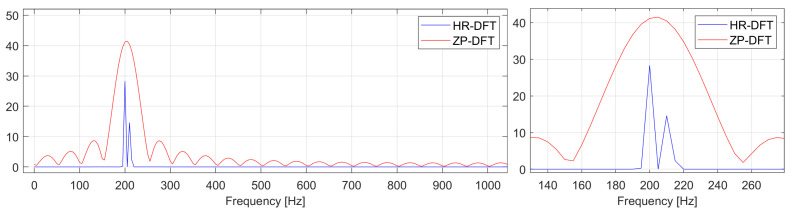
Amplitude spectrum of the signal from Figure 2: (**Left**) Low-resolution DFT (standard DFT) of zero-padded signal (red) and high-resolution DFT (blue) obtained using the above algorithm (10 iterations, 300-point DFT). (**Right**) A close-up of the left plot. Resolution is 5 Hz.

As an example, we will consider the signal composed of two complex harmonics (see Figure 2):

(34) $$x\left(t\right)=e^{j2\pi f_{1}t}+e^{j2\pi f_{2}t},$$

where $f_{1}=200$ Hz and $f_{2}=220$ Hz. The signal is sampled uniformly with a sampling frequency $F_{S}=1005$ Hz and the number of samples generated is N=11. The high-resolution DFT (blue) and the low-resolution DFT (red) of this signal are shown in Figure 3. The length of both DFTs is M=201. The value of $F_{S}$ may appear strange, but it was chosen so that the frequency domain resolution $f_{0}=F_{S}/M=1005/201=5$ Hz is an integer. The number of iterations required to find the high-resolution DFT (in this example) is 10.

The presented example shows the behavior of the proposed algorithm after 10 iterations. The reader can find how the spectrum evolves after each iteration in [19], where other interesting examples, including extrapolation and the reconstruction of example signals, were also given.

**Figure 4:**
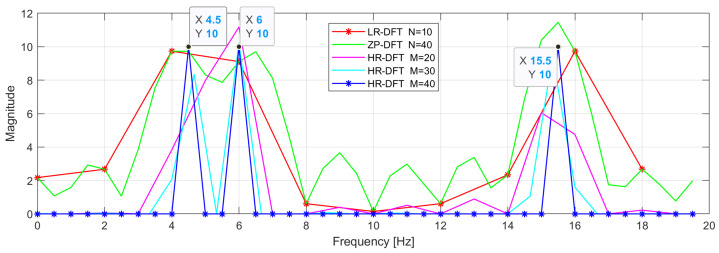
The evolution of the amplitude spectrum of the example signal consisting of 3 complex exponentials with $f_{1}=4.5$ Hz, $f_{2}=6$ Hz, and $f_{3}=15.5$ Hz and amplitudes $A_{1}=A_{2}=A_{3}=1$. The plots show how the spectrum evolves when the length *M* of the high-resolution DFT increases. The length of the signal is N=10 samples. Sampling frequency $F_{S}=20$ Hz.

Here, we want to provide the reader with an example not given in [19] and show how the spectrum evolves when the high-resolution transform length is successively increased. In the example shown in Figure 4, 10 samples of the sum of 3 complex exponentials are transformed using a standard DFT (LR-DFT) of length 10, a zero-padded DFT of length 40, and a high-resolution DFT (HR-DFT) of different lengths: 20, 30, and 40, respectively. As expected, for M=40, when the resolution is 0.5 Hz, the given exponents are found perfectly (without leakage); and in contrast to the case of LR-DFT, where the resolution is 2 Hz, here, the frequencies $f_{1}$ and $f_{2}$ are resolved.

This example shows that the high-resolution transform of length *M* of a signal of length *N* can be better than a zero-padded DFT of length *M* of such a signal, but that it cannot be better than the LR-DFT of the signal of length *M*.

Now, having the complex exponentials in the frequency domain, instead of in the time domain, we only need to invert the time axis of the resulting transform according to (27). This will constitute a high-resolution series with several “spectral” lines (in the time domain), with the first one—exhibiting the shortest time—corresponding to the LOS distance—or 2 × LOS distance—in case of a reflection of the LOS signal to the transmitter (initiator).

## 3. Results

**Figure 5:**
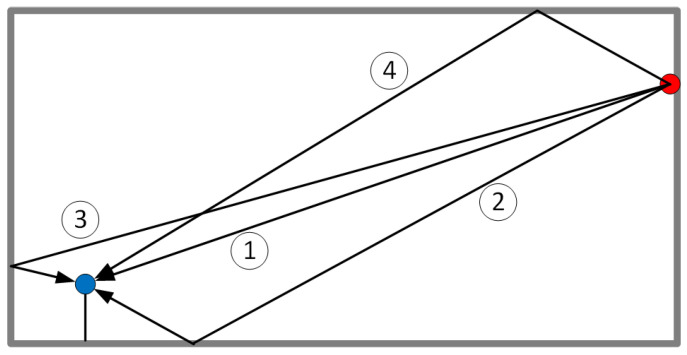
A real 1D stage (linear floor along the bottom line) with width = 20 m and height = 10 m. The red transceiver transmits signals to the blue transceiver. The real path lengths are $d_{1}=15.81$ m (LOS), $d_{2}=16.55$ m, $d_{3}=19.51$ m, and $d_{4}=25.49$ m.

**Figure 6:**
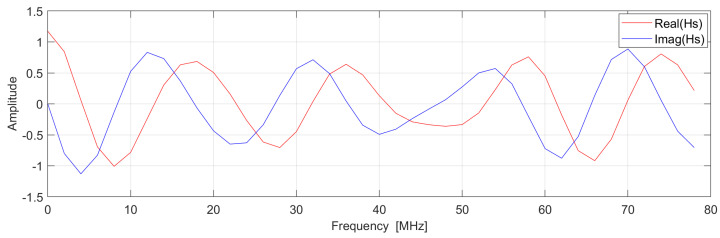
Complex exponentials generated by using example phase data (40 samples, 2 MHz-wide Channel, SNR = 10 dB): real (red) and imaginary (blue) part of a signal consisting of four complex exponentials corresponding to four different paths of the carriers transmitted in 40 2 MHz-wide channels.

One of the relatively new applications of locating objects/people is in virtual acoustics. In this application, we want to know the position of people on a stage so that we can correctly place acoustic objects corresponding to real objects in the virtual acoustic scene. Let us imagine a linear (1D) stage with a width of 20 m and a height of 10 m (for simplicity, we assume that the stage’s depth is 0 m, i.e., we consider a 1D stage) as shown in Figure 5. On this stage, we place an object (an actor) with a transceiver attached (the blue ball). The second transceiver (the red ball) is installed on the right wall. In the BLE standard, the red transceiver usually acts as the Initiator, which sends signals to the blue transceiver (the Reflector), which then sends the received signals back to the Initiator. However, for simplicity, we assume that the transceivers are synchronized, meaning that we can measure the phase of the signal sent from one of those transceivers to the other without sending the received signal back to the transmitter. We assume the radio system is similar to the one in the BLE standard, i.e., we have 40 channels, each 2 MHz wide, in the 2400–2480 MHz band. We consider the LOS path and only one reflection per surface, i.e., three reflections. In Figure 6, we show an example of complex exponentials (Hs) that have been generated using the phase data achieved by simulating the case shown in Figure 5.

**Figure 7:**
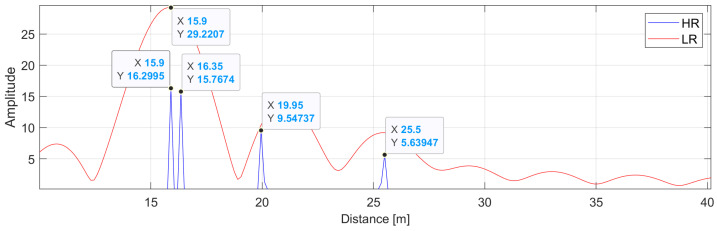
Amplitude spectrum of the phase-signal from Figure 6: (RED) Low-resolution zero-padded IDFT. (BLUE) High-resolution IDFT. The ground truth distances are $d_{1}=15.81$ m, $d_{2}=16.55$ m, $d_{3}=19.85$ m, and $d_{4}=25.49$ m, respectively. SNR = 80 dB.

The result of transforming these signals using high- and low-resolution IDFTs is shown in Figure 7.

**Figure 8:**
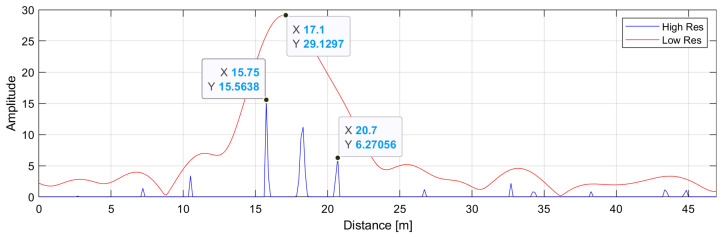
Amplitude spectrum of a phase-signal similar to that from Figure 6: (RED) Low-resolution zero-padded IDFT. (BLUE) High-resolution IDFT. The ground truth distances are $d_{1}=15.81$ m, $d_{2}=17.15$ m, $d_{3}=18.05$ m, and $d_{4}=19.10$ m, respectively. SNR = 20 dB.

In this case, both the standard DFT and high-resolution DFT give good results. However, if the reflections were arranged more evenly and—in addition to that—were noisy, the result might look like that shown in Figure 8. Now, the energy of the transforms is distributed more evenly, causing the shift of the main lobe of the LR DFT towards longer reflections, thus indicating a distance greater than the actual distance. One can also notice several small spurious peaks coming from the noise energy that is falsely focused in those bins. They are more and more evident with the increasing level of noise energy.

**Figure 9:**
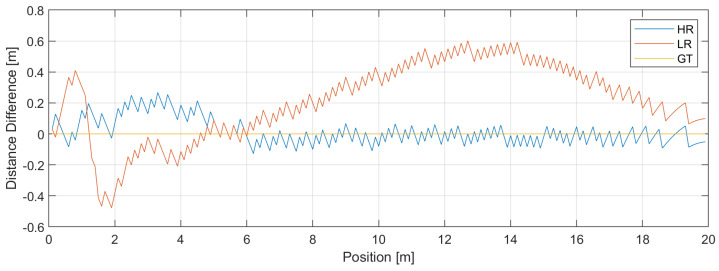
Distance measurement error for high- and low-resolution IDFTs. The distance between the two transceivers has been measured when one of these transceivers (the blue ball) moves along the stage from left to right (see Figure 5). The real distance has been subtracted from the estimated distances. SNR = 80 dB.

These examples show that the results may vary depending on the current distribution of the transform’s energy. This variety is shown in Figure 9, where we show the magnitudes of the HR and LR transforms’ errors when the object moves along the scene from left to right.

As you can see, the error for the LR transform is up to 0.5 m, while the error for the HR transform—in most cases—does not exceed 0.2 m. The mean value and the standard deviation for HR transform are 0.015 m and 0.085 m, while for LR transform, it is 0.213 m and 0.248 m, respectively.

## 4. Discussion

The algorithm proposed here for measuring distances—or rather, for measuring time delays—was originally developed to determine the spectral lines of signals too short to be analyzed by other methods with sufficient resolution. After exchanging the time and frequency variables and reinterpreting the result, it can be used to determine spectral lines in the time domain. In most analyzed cases, the algorithm works correctly and is relatively accurate, although it depends on the number, power, and distribution of spectral lines’ positions. This is visible in Figure 9, where the LR IDFT result, i.e., IDFT of zero-padded series, is stable even though its error is approximately 0.5 m, while this error for the HR transform, with the assumed parameters, is much smaller. Note that HR transform gives results with a relatively small mean error; the error has relatively evenly positive and negative values. In the case of LR transform, most results are positive values. This comes from the specific distribution of the delays; if relatively large energy of the delays is concentrated within the length of the LR IDFT lobe, it can move the center of this lobe to the right from the position of the first delay. On the other hand, the neighboring peaks of the HR transform can “glue” together, forming a peak greater than the first peak (related to LOS delay). This suggests looking for “the first significant” maximum rather than for the maximum, but even then, choosing the right delay, which is beyond the scope of this paper, might be an issue if the SNR drops to roughly below 10dB. As an example of such a procedure, we assumed that the correct delay is the one lying as the first on the left within the lobe, where the LR result, which seems to be stable, suggests its approximate position.

As the proposed algorithm has some parameters to set up, it is difficult to determine how good or bad it is. Nevertheless, a couple of statements seem to be true:

- The length of the HR transform (i.e., the resolution, which is roughly the reciprocal of that length) can be set up dynamically so that the relative error of distance estimation can be approximately the same along the whole path when approaching the asset (the reflector). On the other hand, there are applications, like those presented in the examples given, where we were keeping this resolution constant, though arbitrarily chosen.
- There are fast solutions to (33) that can be realized using Levinson recursion or Cholesky decomposition in the case of non-uniformly distributed data.
- Additionally, the algorithm is constrained on data, so it gives a full transform (and not only magnitude).
- The disadvantage of this algorithm is that in addition to the correct spectral lines, it also “finds” spectral lines in places where they do not exist. This is due to the form of the regularizer, which “prefers” the sparse spectrum. Therefore, if the spectrum contains a “false” energy in a wide band, the algorithm tends to concentrate this energy in a narrow band, thus creating false spectral lines. However, this does not matter much in our case, since this situation usually occurs at a sufficient distance from the first delay, where the spectrum is “continuous” due to noise and possibly many loosely scattered, irrelevant reflections.

We investigated the applicability of the proposed algorithm to the distance estimation task; however, the high, achievable resolution may be a good point of suggestion to consider its use in other applications such as DOA or localization applications, including virtual acoustics, as an example.

## Data Availability

Data are contained within this article.

## Abbreviations

The following abbreviations are used in this manuscript:

PBR	Phase-Based Ranging
BLE	Bluetooth Low Energy
DE	Distance Estimation
DF	Direction Finding
DOA	Direction of Arrival
LOS	Line of Sight
NLOS	Non-Line of Sight

## Appendix A

The physical principle of distance measurement using wavelengths is that the distance between two points that has to be measured can be expressed in a (fractional) number $\alpha_{n}$ of wavelengths of a wave transmitted from one of these points (a transmitter) to the other point (a receiver), that is, for a certain wavelength $\lambda_{n}$:

(A1) $$d=\alpha_{n}\lambda_{n},\;\;\;\;n=1...N\geq 1,$$

where *N* is an arbitrarily selected number of waves of different lengths. From a purely metrological point of view, N=1 is enough, but other issues require using more than one wavelength. If a wave travels from a transmitter (T) to a receiver (R) its argument (phase) changes at the point of T from 0 to ϕ when the wavefront arrives at R; so, we can write

(A2) $$\phi_{n}=2\pi \frac{d}{\lambda_{n}}$$

and because the wave travels with a given speed *c*, we can write

(A3) $$\phi_{n}=2\pi \frac{c}{\lambda_{n}}t=2\pi f_{n}t=\omega_{n}t,\;\;\;\;n=1..N,$$

where *t* is the time of flight of the wave with frequency $f_{n}$ from T to R.

At the same time, we must note that the wave itself is considered a periodic function (real or complex) of the real argument (phase) such that two such functions, with a phase difference of 2π or a multiplicity thereof, are equal to each other, i.e.,

(A4) $$e^{j\phi }=e^{j(\phi +2\pi )}=e^{j(\phi +2\pi m)},\;\;\;\;\;\mathrm{for}\;\mathrm{all}\;\mathrm{integer}\;\;m.$$

This means that this phase can be expressed as follows:

(A5) $$\phi^{\left(u\right)}=\phi^{\left(w\right)}+2\pi M,$$

where $\phi^{\left(w\right)}$ and $\phi^{\left(u\right)}$ are the so-called *wrapped* and *unwrapped* phases, respectively. The value *M* denotes the number of full wavelengths of length λ falling at the considered distance *d*. The principle of measuring the unwrapped (absolute) phase is theoretically quite simple, but in practice, it is useless because it requires counting the number of complete wave periods, which—in turn—requires the synchronization of the transmitter and the receiver. It can also require additional hardware solutions. Instead, knowing from (A3) that $\phi_{n}$ is a linear function of $\omega_{n}$, we can express the wave’s time of flight as

(A6) $$t=\frac{\Delta \phi }{\Delta \omega }=\frac{\phi_{m}^{\left(u\right)}-\phi_{n}^{\left(u\right)}}{\omega_{m}-\omega_{n}},\;\;\;\;\;m,n=1..N,m\neq n.$$

To avoid phase ambiguity during phase unwrapping, the following condition must be true:

(A7) $$|\phi_{n}-\phi_{n-1}|<2\pi .$$

The distance traveled by the wave is d=ct, where *c* is the speed of the wave. This simple phase-based distance measurement method only works well for a single delay. For more than one delay, the phase is no longer the linear function of frequency.
